# Molecular Pathogenesis of Pancreatic Neuroendocrine Tumors

**DOI:** 10.3390/cancers2041901

**Published:** 2010-11-18

**Authors:** Florian Ehehalt, Ellen Franke, Christian Pilarsky, Robert Grützmann

**Affiliations:** Department for General, Thoracic and Vascular Surgery, University Hospital “Carl Gustav Carus”, University of Technology, Dresden, Germany; E-Mails: frankeellen@web.de (E.F.); christian.pilarsky@uniklinikum-dresden.de (C.P); robert.gruetzmann@uniklinikum-dresden.de (R.G.)

**Keywords:** pancreatic neuroendocrine tumors, genetics, pathogenesis, menin, MEN-1, VHL, NF-1, mTOR

## Abstract

Pancreatic neuroendocrine tumors (PNETs) are rare primary neoplasms of the pancreas and arise sporadically or in the context of genetically determined syndromes. Depending on hormone production and sensing, PNETs clinically manifest due to a hormone-related syndrome (functional PNET) or by symptoms related to tumor bulk effects (non-functional PNET). So far, radical surgical excision is the only therapy to cure the disease. Development of tailored non-surgical approaches has been impeded by the lack of experimental laboratory models and there is, therefore, a limited understanding of the complex cellular and molecular biology of this heterogeneous group of neoplasm. This review aims to summarize current knowledge of tumorigenesis of familial and sporadic PNETs on a cellular and molecular level. Open questions in the field of PNET research are discussed with specific emphasis on the relevance of disease management.

## 1. Introduction

PNETs are a heterogeneous group of pancreatic primaries characterized by expression of proteins associated with the secretory vesicles (neuron-specific enolase, synaptophysin and/or chromogranin A). Functional tumors, such as insulinomas or gastrinomas, are specified by a clinical syndrome (e.g., Whipple trias, Zollinger-Ellison-syndrome *etc*.), which can be attributed to hormone excess. Non‑functional tumors—although they do in some cases produce hormones (e.g., pancreatic polypeptide)—by definition do not result in a clinical syndrome. If not sporadic, PNETs are part of genetically determined syndromes, for example multiple endocrine neoplasia 1 (MEN-1).

**Figure 1:**
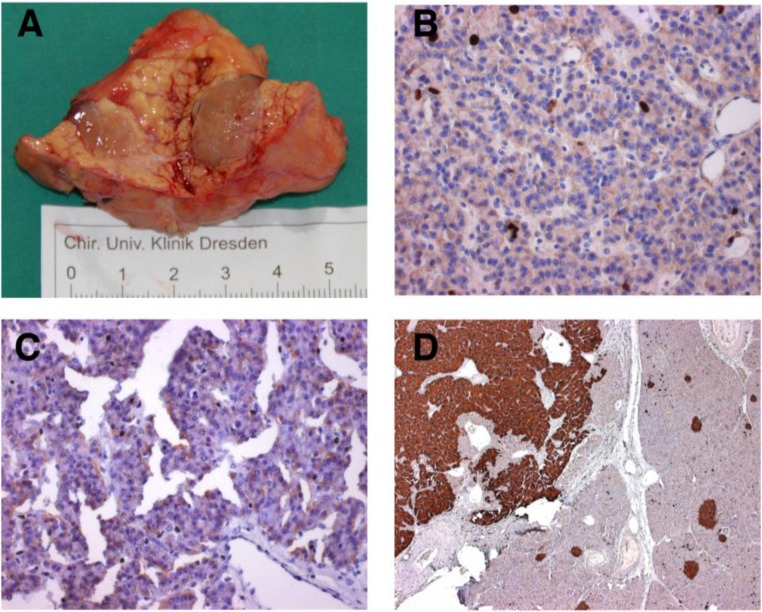
(**A**) Enucleated pancreatic insulinoma; (**B**) immunostaining of the insulinoma revealed Ki-67 expression (brown) <2%; (**C**) weak insulin expression (brown) and (**D**) strong expression of synaptophysin (brown).

PNETs are rare tumors, accounting for <3% of all pancreatic neoplasms [1]. The annual incidence is estimated to be 2.2 in 1.000.000 [2]. Five-year overall survival after resection is strikingly associated with tumor type and ranges from 97% for insulinomas (Figure 1) to 30% for non-functional PNETs [3] and is superior to the prognosis of pancreatic ductal adenocarcinomas. In recent studies, 68–90% of PNETs were classified to be non-functional [4].

The rarity of the disease, its heterogeneous endocrinological and oncological behavior, and the lack of appropriate research models aggravate investigation of the molecular pathogenesis of PNETs [5]. Nevertheless, one specific molecular characteristic of selected PNETs, *i.e.*, expression of somatostatin receptor subtypes 2 and 5, is already well established in diagnosis and therapy, since radioisotopes bound to somatostatin analogues facilitate localization and staging while somatostatin receptor agonists play a role in symptom control and chemotherapy of these tumors [4].

Genetically determined syndromes and high-throughput analyses of sporadic PNETs can shed light on possible molecular hallmarks of tumor initiation. This review is intended to collect the latest data about PNET’s molecular characteristics and their implications on pathogenesis and therapy of these tumors.

## 2. Methods

PubMed/Medline and Google Scholar databases served for selective literature research. “Pancreatic neuroendocrine tumor” or “insulinoma” or “gastrinoma” were used as queries. Search results were screened for contents covering aspects of molecular pathogenesis of the disease. The citations of the selected papers were used for acquisition of additional relevant publications.

## 3. Review of Literature

### 3.1. MEN-1 Syndrome and the Role of Menin in PNET Initiation

The tumor suppressor gene MEN-1 and its protein product menin appear to be the most extensively investigated factor involved in the development of PNETs. For MEN-1 syndrome, more than 300 germline mutations were reported which result in loss-of-function of menin [6]. The tumor suppressing function of menin is not fully understood. However, regulation of gene transcription, genome stability, cell proliferation and apoptosis, were attributed to the menin function (Table 1) [7].

**Table 1:** Mechanisms of menin tumor suppression (according to [7]).

Mechanism	Co-factor	Regulated Factor	Consequence
Transcription activation	HMT	p27^kip1^	Cell growth inhibition
		p18^ink4c^	
		Hoxc8	Cell differentiation
Transcription repression	HDAC	IGFBP-2	Decreased cell proliferation
Inhibition	?	Cyclin D/CDK4	Inhibition of G1/S transition
		cdc7/ASK	Inhibition of DNA synthesis
Transcription activation	?	Caspase 8	TNFα- sensitizing/apoptosis
Protein-protein interactions	FancD2/RPA2/ cdc7/ASK	hTERT	Genome stabilization

Abbreviations: HMT: histone methyl transferase; HDAC: histone deacetylase; FancD2: Fanconi anemia group D2 protein; RPA: replication protein; CDC: cell division cycle; ASK: activator of S-phase kinase; Hox: homeobox gene; IGFBP: insulin-like growth factor binding protein; CDK: cyclin dependent kinase; hTERT: telomerase reverse transcriptase

Germline loss-of-function *MEN-1* mutation leads to the formation of numerous microadenomas, mostly resulting in non-functional PNET and insulinomas [8], while MEN-1 associated gastrinomas are regularly located within the duodenum [9]. The *MEN-1* gene is located on the chromosome 11q13. Loss of heterozygosity of 11q or somatic *MEN-1* mutation is present in up to 46% of sporadic PNETs independent of tumor stage, which make menin and/or other tumor suppressors on 11q good candidates to be involved in tumor initiation in PNETs [10,11,12]. Subcellular distribution of menin is disturbed in 80% of sporadic PNETs [13].

While heterozygous knock-out of *MEN-1* in mice results in a good model for the disease [14], the complexity of menin function was recently underlined by the investigation of rodent islets after α-cell specific knock out of *MEN-1*, which resulted in the formation of glucagonomas and insulinomas. Trans-differentiation of α- into β-cells and, consecutively, the development of insulinomas were observed. The authors conclude that, besides its tumor suppressing function, menin is a regulator of endocrine cell plasticity. Its disruption in one of the endocrine cell populations is sufficient for tumor initiation and may result in hormone secretion different from the original mutation-bearing cell [15]. This data might, at least partially, explain non-endocrine precursor lesions of PNETs observed in pancreatic tissue of MEN-1 patients [16].

Aside from MEN-1, other genetic syndromes can lead to PNETs. Specifically, von Hippel-Lindau disease (VHL), neurofibromatosis type 1 (NF-1) and possibly tuberous sclerosis (TSC) are associated with endocrine neoplasms.

### 3.2. Role of Angiogensis in PNET Progression

Although the exact mechanisms of the PNET development in VHL patients are not yet elucidated, dysregulated neogenesis of vessels is likely to be one of the key mechanisms. The penetrance of *VHL* mutation on the development of PNETs is low. Only 11–17% of VHL patients develop true endocrine neoplasia of the pancreas [17,18]. The *VHL* gene is located on chromosome 3p25–26 [19]. Its germline mutations are highly heterogenous and result in the loss-of-function of the protein [20]. The VHL protein is known to degrade the alpha-subunit of hypoxia-inducible factor (HIF). Lack of HIF degradation leads to the uncontrolled production of factors associated with vessel neogenesis [21]. According to this mechanism, PNETs are highly vascularized tumors, thus distinguishable from other pancreatic primaries [22]. Allelic loss centromeric to *VHL* locus was observed in VHL kindred and loss of heterozygosity on chromosme 3p occurs subsequently to *VHL* mutation and correlates with malignant progression, suggesting a stepwise genetic progression of the disease [12,23,24].

Loss of heterozygosity on chromosome 3p is present in only 30% of sporadic PNETs and is not associated with somatic mutations of VHL. However, the pivotal role of pathological angiogenesis for tumor progression in sporadic PNETs was emphasized by several recent studies. Hypervascularization, tumor architecture and CXCL-12 (chemokine (C-X-C motif) ligand 12) expression are prognostic features of PNETs [25]. Among other thyrosine kinase receptors, epidermal growth factor receptor (EGFR) [26] and vascular endothelial growth factor receptor 2 (VEGFR-2) [27] are likely to be involved in PNET’s neo-angiogenesis in humans. There is good evidence, that angiopoietin-2 promotes tumor progression [28]. A rodent multi-stage PNET model revealed the loss of the cell cycle regulator p19^ARF^ to facilitate the angiogenic switch and tumor initiation of PNETs [29].

The clinical relevance of PNET’s deranged vascularization arises from new and promising therapeutic anti-angiogenetic approaches under investigation in phase II and III clinical trials [30].

### 3.3. Dysregulated Cell Growth and Proliferation in PNETs

NF-1 and TSC are genetically determined diseases resulting in different tumor syndromes. The association of NF-1 and TSC with duodenal and pancreatic endocrine tumors was casually reported [31,32,33,34,35].

*NF-1* or *TSC1/2* mutations result in loss of function of their protein products neurofibromin and tuberin, respectively. Notably, the intact proteins suppress the function of a common target, namely mTOR (mammalian target of rapamycin) [36,37]. Furthermore, hypoxia- and HIF-dependent mTOR activation links disturbed mTOR signaling to VHL disease [38]. mTOR is a key regulator of cell growth and integrates a wide variety of cellular inputs, such as growth factors, nutrients, energy status and hypoxia-induced stress. For example, insulin like growth factor receptor type 1 (IGF-1R) is a tyrosine kinase upstream of mTOR. In a rodent model, it was shown that IGF-1R functionality is positively correlated with tumor progression [39].

**Figure 2:**
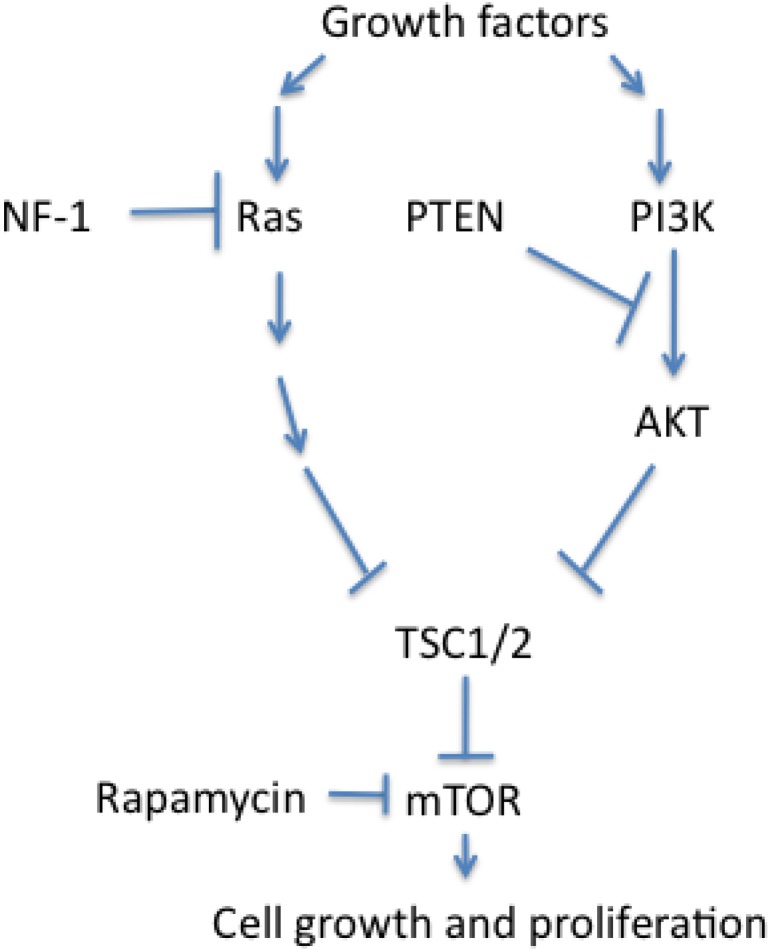
Scheme of mTOR-signaling pathway.

mTOR activation leads to a myriad of cell responses and culminates in uncontrolled cell growth. It can be pharmacologically suppressed by rapamycin (Figure 2) [40]. Thus, mTOR shows signs of being a formidable candidate for a therapeutic approach in PNETs.

Over the last years, high-throughput analyses of differential gene expression of sporadic and MEN-1 associated PNETs yielded several biomarkers, specifically for tumor progression (metastasis) and differentiation from other neuroendocrine cancers [41,42,43,44,45,46,47]. However, none of these biomarkers was sufficient to correlate with patient’s outcome or therapeutic impact. Most likely, patient input (8–12 subjects) was too low and study designs varied widely. Recently, the lack of insufficient case‑load was overcome by Missiaglia and colleagues, who recruited 72 sporadic primary PNETs, seven matched metastases, five normal pancreata and five islet preparations for differential gene expression analysis. Notably, the study was able to show that two important inhibitors of the mTOR-pathway, specifically TSC2 and Phosphatase and Tensin Homolog protein (PTEN), were down-regulated in PNETs and, on univariate analysis, inversely correlated with tumor aggressiveness and prognosis. The resulting activation of the mTOR-pathway could be suppressed by rapamycin in PNET cell lines. Additionally, the data was sufficient to show that the expression level of fibroblast growth factor 13 in PNETs correlates with poor prognosis [48].

These data serve as a rationale for treatment of advanced PNETs with Rapamycin and its analogues. So far, two clinical trials evaluating rapamycin and analogues recently raised hope for efficient disease control [49,50,51]. Compensatory activation of AKT might be overcome by a combination of therapies [52].

## 4. Conclusions

Although the complex natural history and rarity of PNETs has hampered investigation of tumor initiation and progression for a long time, recent studies on genetically determined and sporadic disease, as well as the establishment of new animal models and cell lines, shed some light on the underlying mechanisms. Noteworthy, a remarkable body of data points to the mTOR-signaling pathway to be centrally dysregulated in PNETs due to several mechanisms. This fact is already reflected in clinically tested mTOR suppressive therapies. Furthermore, mechanisms of dysregulated hypervascularization of the tumor are now at least partially understood. The relevance of tumor neo‑angiogenesis on progression and prognosis was perceived and is actually under investigation in interventional clinical trials.

How far epigenetic modifications [53] and differential microRNA-expression [54] are mechanistically involved in the dysregulated signaling pathways of PNETs remains a matter for further investigation. Large tumor banks and significant animal models are desired for additional studies.

## References

[1] Yao J.C., Eisner M.P., Leary C., Dagohoy C., Phan A., Rashid A., Hassan M., Evans D.B. (2007). Population-based study of islet cell carcinoma. Ann. Surg. Oncol..

[2] Halfdanarson T.R., Rabe K.G., Rubin J., Petersen G.M. (2008). Pancreatic neuroendocrine tumors (pnets): Incidence, prognosis and recent trend toward improved survival. Ann. Oncol..

[3] Mansour J.C., Chen H. (2004). Pancreatic endocrine tumors. J. Surg. Res..

[4] Ehehalt F., Saeger H.D., Schmidt C.M., Grützmann R. (2009). Neuroendocrine tumors of the pancreas. Oncologist.

[5] Modlin I.M., Moss S.F., Chung D.C., Jensen R.T., Snyderwine E. (2008). Priorities for improving the management of gastroenteropancreatic neuroendocrine tumors. J. Natl. Cancer. Inst..

[6] Leotlela P.D., Jauch A., Holtgreve-Grez H., Thakker R.V. (2003). Genetics of neuroendocrine and carcinoid tumours. Endocr. Relat. Cancer.

[7] Yang Y., Hua X. (2007). In search of tumor suppressing functions of menin. Mol. Cell. Endocrinol..

[8] Akerstrom G., Hessman O., Hellman P., Skogseid B. (2005). Pancreatic tumours as part of the men-1 syndrome. Best Pract. Res. Clin. Gastroenterol..

[9] Anlauf M., Garbrecht N., Henopp T., Schmitt A., Schlenger R., Raffel A., Krausch M., Gimm O., Eisenberger C.F., Knoefel W.T., Dralle H., Komminoth P., Heitz P.U., Perren A., Kloppel G. (2006). Sporadic versus hereditary gastrinomas of the duodenum and pancreas: Distinct clinico-pathological and epidemiological features. World J. Gastroenterol..

[10] Shan L., Nakamura Y., Nakamura M., Yokoi T., Tsujimoto M., Arima R., Kameya T., Kakudo K. (1998). Somatic mutations of multiple endocrine neoplasia type 1 gene in the sporadic endocrine tumors. Lab. Invest..

[11] Wang E.H., Ebrahimi S.A., Wu A.Y., Kashefi C., Passaro E., Sawicki M.P. (1998). Mutation of the menin gene in sporadic pancreatic endocrine tumors. Cancer Res..

[12] Oberg K. (2009). Genetics and molecular pathology of neuroendocrine gastrointestinal and pancreatic tumors (gastroenteropancreatic neuroendocrine tumors). Curr. Opin. Endocrinol. Diabetes Obes..

[13] Corbo V., Dalai I., Scardoni M., Barbi S., Beghelli S., Bersani S., Albarello L., Doglioni C., Schott C., Capelli P., Chilosi M., Boninsegna L., Becker K.F., Falconi M., Scarpa A. Men1 in pancreatic endocrine tumors: Analysis of gene and protein status in 169 sporadic neoplasms reveals alterations in the vast majority of cases. Endocr. Relat. Cancer.

[14] Bertolino P., Tong W.M., Galendo D., Wang Z.Q., Zhang C.X. (2003). Heterozygous men1 mutant mice develop a range of endocrine tumors mimicking multiple endocrine neoplasia type 1. Mol. Endocrinol..

[15] Lu J., Herrera P.L., Carreira C., Bonnavion R., Seigne C., Calender A., Bertolino P., Zhang C.X. Alpha cell-specific men1 ablation triggers the transdifferentiation of glucagon-expressing cells and insulinoma development. Gastroenterology.

[16] Vortmeyer A.O., Huang S., Lubensky I., Zhuang Z. (2004). Non-islet origin of pancreatic islet cell tumors. J. Clin. Endocrinol. Metab..

[17] Corcos O., Couvelard A., Giraud S., Vullierme M.P., Dermot O.T., Rebours V., Stievenart J.L., Penfornis A., Niccoli-Sire P., Baudin E., Sauvanet A., Levy P., Ruszniewski P., Richard S., Hammel P. (2008). Endocrine pancreatic tumors in von hippel-lindau disease: Clinical, histological, and genetic features. Pancreas.

[18] Lubensky I.A., Pack S., Ault D., Vortmeyer A.O., Libutti S.K., Choyke P.L., Walther M.M., Linehan W.M., Zhuang Z. (1998). Multiple neuroendocrine tumors of the pancreas in von hippel-lindau disease patients: Histopathological and molecular genetic analysis. Am. J. Pathol..

[19] Latif F., Tory K., Gnarra J., Yao M., Duh F.M., Orcutt M.L., Stackhouse T., Kuzmin I., Modi W., Geil L. (1993). Identification of the von hippel-lindau disease tumor suppressor gene. Science.

[20] Lonser R.R., Glenn G.M., Walther M., Chew E.Y., Libutti S.K., Linehan W.M., Oldfield E.H. (2003). Von hippel-lindau disease. Lancet.

[21] Kaelin W.G. (2002). Molecular basis of the vhl hereditary cancer syndrome. Nat. Rev. Cancer.

[22] Kersting S., Konopke R., Kersting F., Volk A., Distler M., Bergert H., Saeger H.D., Grützmann R., Bunk A. (2009). Quantitative perfusion analysis of transabdominal contrast-enhanced ultrasonography of pancreatic masses and carcinomas. Gastroenterology.

[23] Lott S.T., Chandler D.S., Curley S.A., Foster C.J., El-Naggar A., Frazier M., Strong L.C., Lovell M., Killary A.M. (2002). High frequency loss of heterozygosity in von hippel-lindau (vhl)-associated and sporadic pancreatic islet cell tumors: Evidence for a stepwise mechanism for malignant conversion in vhl tumorigenesis. Cancer Res..

[24] Starker L.F., Carling T. (2009). Molecular genetics of gastroenteropancreatic neuroendocrine tumors. Curr. Opin. Oncol..

[25] Takahashi Y., Akishima-Fukasawa Y., Kobayashi N., Sano T., Kosuge T., Nimura Y., Kanai Y., Hiraoka N. (2007). Prognostic value of tumor architecture, tumor-associated vascular characteristics, and expression of angiogenic molecules in pancreatic endocrine tumors. Clin. Cancer Res..

[26] Papouchado B., Erickson L.A., Rohlinger A.L., Hobday T.J., Erlichman C., Ames M.M., Lloyd R.V. (2005). Epidermal growth factor receptor and activated epidermal growth factor receptor expression in gastrointestinal carcinoids and pancreatic endocrine carcinomas. Mod. Pathol..

[27] Silva S.R., Bowen K.A., Rychahou P.G., Jackson L.N., Weiss H.L., Lee E.Y., Townsend C.M., Evers B.M. (2010). Vegfr-2 expression in carcinoid cancer cells and its role in tumor growth and metastasis. Int. J. Cancer.

[28] Detjen K.M., Rieke S., Deters A., Schulz P., Rexin A., Vollmer S., Hauff P., Wiedenmann B., Pavel M., Scholz A. (2010). Angiopoietin-2 promotes disease progression of neuroendocrine tumors. Clin. Cancer Res..

[29] Ulanet D.B., Hanahan D. (2010). Loss of p19(arf) facilitates the angiogenic switch and tumor initiation in a multi-stage cancer model via p53-dependent and independent mechanisms. PLoS One.

[30] Eriksson B. (2010). New drugs in neuroendocrine tumors: Rising of new therapeutic philosophies?. Curr. Opin. Oncol..

[31] Francalanci P., Diomedi-Camassei F., Purificato C., Santorelli F.M., Giannotti A., Dominici C., Inserra A., Boldrini R. (2003). Malignant pancreatic endocrine tumor in a child with tuberous sclerosis. Am. J. Surg. Pathol..

[32] Eledrisi M.S., Stuart C.A., Alshanti M. (2002). Insulinoma in a patient with tuberous sclerosis: Is there an association?. Endocr. Pract..

[33] Verhoef S., van Diemen-Steenvoorde R., Akkersdijk W.L., Bax N.M., Ariyurek Y., Hermans C.J., van Nieuwenhuizen O., Nikkels P.G., Lindhout D., Halley D.J., Lips K., van den Ouweland A.M. (1999). Malignant pancreatic tumour within the spectrum of tuberous sclerosis complex in childhood. Eur. J. Pediatr..

[34] Cappelli C., Agosti B., Braga M., Cumetti D., Gandossi E., Rizzoni D., Agabiti Rosei E. (2004). Von recklinghausen's neurofibromatosis associated with duodenal somatostatinoma. A case report and review of the literature. Minerva Endocrinol..

[35] Tan C.C., Hall R.I., Semeraro D., Irons R.P., Freeman J.G. (1996). Ampullary somatostatinoma associated with von recklinghausen's neurofibromatosis presenting as obstructive jaundice. Eur. J. Surg. Oncol..

[36] Inoki K., Corradetti M.N., Guan K.L. (2005). Dysregulation of the tsc-mtor pathway in human disease. Nat. Genet..

[37] Johannessen C.M., Reczek E.E., James M.F., Brems H., Legius E., Cichowski K. (2005). The nf1 tumor suppressor critically regulates tsc2 and mtor. Proc. Natl. Acad. Sci. USA.

[38] Brugarolas J., Lei K., Hurley R.L., Manning B.D., Reiling J.H., Hafen E., Witters L.A., Ellisen L.W., Kaelin W.G. (2004). Regulation of mtor function in response to hypoxia by redd1 and the tsc1/tsc2 tumor suppressor complex. Genes Dev..

[39] Ulanet D.B., Ludwig D.L., Kahn C.R., Hanahan D. (2010). Insulin receptor functionally enhances multistage tumor progression and conveys intrinsic resistance to igf-1r targeted therapy. Proc. Natl. Acad. Sci. USA.

[40] Sarbassov D.D., Ali S.M., Sabatini D.M. (2005). Growing roles for the mtor pathway. Curr. Opin. Cell. Biol..

[41] Maitra A., Hansel D.E., Argani P., Ashfaq R., Rahman A., Naji A., Deng S., Geradts J., Hawthorne L., House M.G., Yeo C.J. (2003). Global expression analysis of well-differentiated pancreatic endocrine neoplasms using oligonucleotide microarrays. Clin. Cancer Res..

[42] Hansel D.E., Rahman A., House M., Ashfaq R., Berg K., Yeo C.J., Maitra A. (2004). Met proto-oncogene and insulin-like growth factor binding protein 3 overexpression correlates with metastatic ability in well-differentiated pancreatic endocrine neoplasms. Clin. Cancer Res..

[43] Capurso G., Lattimore S., Crnogorac-Jurcevic T., Panzuto F., Milione M., Bhakta V., Campanini N., Swift S.M., Bordi C., Delle Fave G., Lemoine N.R. (2006). Gene expression profiles of progressive pancreatic endocrine tumours and their liver metastases reveal potential novel markers and therapeutic targets. Endocr. Relat. Cancer.

[44] Couvelard A., Hu J., Steers G., O'Toole D., Sauvanet A., Belghiti J., Bedossa P., Gatter K., Ruszniewski P., Pezzella F. (2006). Identification of potential therapeutic targets by gene-expression profiling in pancreatic endocrine tumors. Gastroenterology.

[45] Bloomston M., Durkin A., Yang I., Rojiani M., Rosemurgy A.S., Enkmann S., Yeatman T.J., Zervos E.E. (2004). Identification of molecular markers specific for pancreatic neuroendocrine tumors by genetic profiling of core biopsies. Ann. Surg. Oncol..

[46] Dilley W.G., Kalyanaraman S., Verma S., Cobb J.P., Laramie J.M., Lairmore T.C. (2005). Global gene expression in neuroendocrine tumors from patients with the men1 syndrome. Mol. Cancer.

[47] Duerr E.M., Mizukami Y., Ng A., Xavier R.J., Kikuchi H., Deshpande V., Warshaw A.L., Glickman J., Kulke M.H., Chung D.C. (2008). Defining molecular classifications and targets in gastroenteropancreatic neuroendocrine tumors through DNA microarray analysis. Endocr. Relat. Cancer.

[48] Missiaglia E., Dalai I., Barbi S., Beghelli S., Falconi M., della Peruta M., Piemonti L., Capurso G., Di Florio A., delle Fave G., Pederzoli P., Croce C.M., Scarpa A. (2010). Pancreatic endocrine tumors: Expression profiling evidences a role for akt-mtor pathway. J. Clin. Oncol..

[49] Duran I., Kortmansky J., Singh D., Hirte H., Kocha W., Goss G., Le L., Oza A., Nicklee T., Ho J., Birle D., Pond G.R., Arboine D., Dancey J., Aviel-Ronen S., Tsao M.S., Hedley D., Siu L.L. (2006). A phase ii clinical and pharmacodynamic study of temsirolimus in advanced neuroendocrine carcinomas. Br. J. Cancer.

[50] O'Donnell P.H., Ratain M.J. (2007). Evaluating the activity of temsirolimus in neuroendocrine cancer. Br. J. Cancer.

[51] Yao J.C., Phan A.T., Chang D.Z., Wolff R.A., Hess K., Gupta S., Jacobs C., Mares J.E., Landgraf A.N., Rashid A., Meric-Bernstam F. (2008). Efficacy of rad001 (everolimus) and octreotide lar in advanced low- to intermediate-grade neuroendocrine tumors: Results of a phase ii study. J. Clin. Oncol..

[52] Zitzmann K., Ruden J., Brand S., Goke B., Lichtl J., Spottl G., Auernhammer C.J. (2010). Compensatory activation of akt in response to mtor and raf inhibitors - a rationale for dual-targeted therapy approaches in neuroendocrine tumor disease. Cancer Lett..

[53] House M.G., Herman J.G., Guo M.Z., Hooker C.M., Schulick R.D., Lillemoe K.D., Cameron J.L., Hruban R.H., Maitra A., Yeo C.J. (2003). Aberrant hypermethylation of tumor suppressor genes in pancreatic endocrine neoplasms. Ann. Surg..

[54] Roldo C., Missiaglia E., Hagan J.P., Falconi M., Capelli P., Bersani S., Calin G.A., Volinia S., Liu C.G., Scarpa A., Croce C.M. (2006). Microrna expression abnormalities in pancreatic endocrine and acinar tumors are associated with distinctive pathologic features and clinical behavior. J. Clin. Oncol..

